# Bridging physics and practice: evaluating sensitivity, septal penetration, and detector dead time in terbium-161 gamma-camera imaging

**DOI:** 10.1186/s40658-025-00792-x

**Published:** 2025-08-28

**Authors:** Frida Westerbergh, Lisa McDougall, Philipp Ritt, Julia G. Fricke, Nicholas P. van der Meulen, Cristina Müller, Roger Schibli, Damian Wild, Peter Bernhardt

**Affiliations:** 1 Department of Medical Radiation Sciences, Institute of Clinical Sciences, Sahlgrenska Academy at University of Gothenburg, Gula Stråket 2B, Gothenburg, 413 45 Sweden; 2 Division of Nuclear Medicine, University Hospital Basel, Basel, Switzerland; 3 ITM Oncologics GmbH, Lichtenbergstrasse 1, 85748 Garching, Munich, Germany; 4 Chair for Clinical Nuclear Medicine, Friedrich-Alexander-Universität Erlangen-Nürnberg, 91054 Erlangen, Germany; 5 Laboratory of Radiochemistry, PSI Center for Nuclear Engineering and Sciences, Villigen-PSI, Villigen, Switzerland; 6 Center for Radiopharmaceutical Sciences, PSI Center for Life Sciences, Villigen-PSI, Villigen, Switzerland; 7 Department of Chemistry and Applied Biosciences, ETH Zurich, Zurich, 8093 Switzerland; 8 Department of Medical Physics and Biomedical Engineering (MFT), Sahlgrenska University Hospital, Gothenburg, Sweden

**Keywords:** Terbium-161, 161Tb, Tb-161, SPECT, Dead time, Gamma camera, Sensitivity, Septal penetration, Collimator, Dosimetry

## Abstract

**Introduction/Aim:**

Terbium-161 (^161^Tb) has emerged as a promising therapeutic radionuclide, yet standardized imaging guidelines are lacking. This study aimed to characterize a SPECT/CT system, currently used in an ongoing clinical trial (BETA PLUS; NCT05359146), focusing on sensitivity, septal penetration, and dead-time effects.

**Methods:**

Measurements were conducted on a Siemens Symbia Intevo system using two collimators: low-energy high-resolution (LEHR) and medium-energy low-penetration (MELP). Two energy windows were evaluated: 75 keV ± 10% and 48 keV ± 20%. Planar sensitivity and penetration were assessed using a ^161^Tb-filled Petri dish. Penetration fractions were determined as a function of distance for each collimator-window combination. Dead time was measured intrinsically for each detector using a set of ^161^Tb point sources. SPECT measurements of a homogenous cylinder phantom were performed to assess count rate performance and predict activity levels at which dead-time effects could occur. To evaluate the potential impact of dead time in patient imaging, SPECT projection data from patients treated with 1 GBq of [^161^Tb]Tb-DOTA-LM3 (*n* = 8) was analyzed.

**Results:**

Sensitivity was comparable for both collimators at 75 keV (LEHR: 15.7 cps/MBq, MELP: 18.5 cps/MBq) and increased at 48 keV (LEHR: 44.4 cps/MBq, MELP: 67.9 cps/MBq). Maximum penetration occurred at 75 keV with the LEHR collimator (7.5% at 10 cm). In acquired spectra, more than half of the detected counts (51.6%) appeared above the 75 keV window with LEHR, compared to only 12.2% with MELP. Dead-time analyses revealed non-linear detector responses at wide-spectrum count rates exceeding 93 kcps, corresponding to in-field activities of 1.4–2.0 GBq for LEHR and 1.7–2.2 GBq for MELP. The dead-time constant was determined to 0.42 µs for both detector heads, however, the maximum recorded count rate differed significantly (384 kcps vs. 546 kcps). The median and maximum wide-spectrum count rate for patients treated with [^161^Tb]Tb-DOTA-LM3 was estimated to ~ 20 and ~ 40 kcps per GBq 3 h p.i., respectively, when imaged with LEHR, corresponding to a maximum estimated dead-time loss of 1.7%.

**Conclusions:**

While high-quality ^161^Tb SPECT imaging is feasible, careful consideration is essential; the wide range of photons emitted will produce a higher wide-spectrum count rate as compared to ^177^Lu. The use of low-energy collimators increases penetration and scatter, impairing quantitative accuracy and elevating the wide-spectrum count rate, which may intensify dead-time effects. At therapeutic activity levels (e.g., 7.4 GBq), dead time should be closely monitored to ensure reliable quantification.

**Supplementary Information:**

The online version contains supplementary material available at 10.1186/s40658-025-00792-x.

## Introduction

In recent years, ^161^Tb has surfaced as a viable therapeutic radionuclide, exhibiting many similarities to the widely adopted ^177^Lu. Both are members of the lanthanide series, decay with a half-life of approximately one week, and emit β^−^ particles with comparable energies [1].

However, ^161^Tb distinguishes itself with a significant co-emission of conversion and Auger electrons. These low-energy emissions are characterized by a short range and higher linear energy transfer, resulting in a more localized energy deposition, which is theorized to enhance effectiveness in treating micrometastases [2, 3]. The benefits of ^161^Tb over ^177^Lu have been demonstrated in vitro and in vivo [4–6]. Currently, ^161^Tb is rapidly advancing into a clinical setting, with ongoing clinical trials for both neuroendocrine tumors (NCT05359146) [7] and advanced prostate cancer (NCT05521412, NCT04833517, and NCT06343038) [8–10].

While the decay characteristics of ^161^Tb are believed to be favorable in terms of therapeutic efficiency, they also present some unique challenges regarding imaging. The most prominent photon emissions—sufficiently abundant for imaging—are low in energy, including the 75 keV γ-emission (10.2% yield) and the 49 keV γ-emission (17.0%), as well as adjacent X-ray emissions at 45.2 keV (6.28%), 46.0 keV (11.2%), and 52.2 keV (3.6%) [11]. While the usual operating range of most NaI(Tl)-based γ-camera systems is above 100 keV, energies as low as ~ 30–40 keV can often be imaged efficiently.

A more significant challenge arises from the very characteristics that make ^161^Tb appealing—the substantial emission of conversion and Auger electrons. The emission of conversion electrons competes with the emission of γ, resulting in an intricate spectrum including of many γ-emissions (33 in total, compared to just 6 for ^177^Lu) over a wide energy range (25.7–550 keV) [11, 12]. Although most of these emissions are low in yield (27 out of 33, < 0.1%), they could contribute to septal penetration and image degradation if not properly addressed.

Similarly, the emission of Auger electrons competes with the emission of X-rays. As a result, ^161^Tb exhibits intense X-ray emissions, with 5 emissions contributing to a combined yield of 44%, compared to just 9% for ^177^Lu [11, 12]. This raises concerns about dead time, as the overall signal produced per unit activity is presumably higher.

All together, these factors contribute to a complex emission spectrum that poses challenges for accurate imaging with ^161^Tb. Due to the novelty of this radionuclide, comprehensive imaging guidelines are lacking. The aim of this study was to characterize the performance of a clinical SPECT/CT system, currently employed in two clinical ^161^Tb trials (BETA PLUS; NCT05359146 and PROGNOSTICS; NCT06343038), with a focus on planar sensitivity, septal penetration, and dead time.

## Methods

### Equipment and general acquisition parameters

**Fig. 1:**
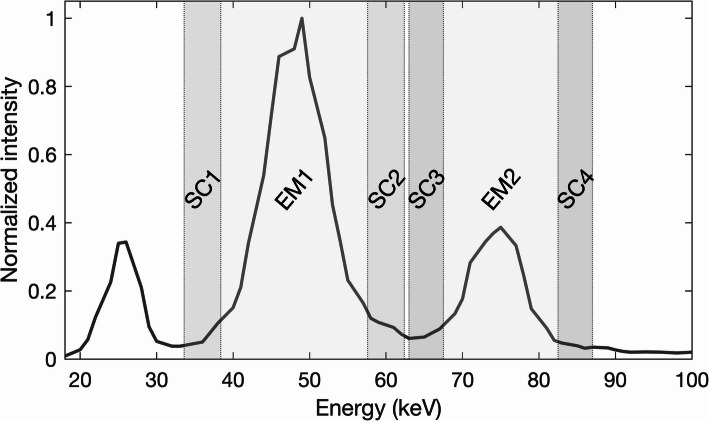
The ^161^Tb energy protocol employed, consisting of two photopeak windows (EM1 and EM2) at 48 keV ± 20% and 75 keV ± 10%, along with four scatter windows (SC1–SC4): 10% for EM1 and 6% for EM2, illustrated on an energy spectra acquired from an in-air measurement of a Petri dish with LEHR collimation (40 cm distance)

Measurements were conducted using a Siemens Symbia Intevo system with a 3/8” NaI(Tl) crystal, and two separate collimators: low-energy high-resolution (LEHR) and medium-energy low-penetration (MELP). The ^161^Tb energy protocol employed consisted of two photopeak windows: 75 keV ± 10% and 48 keV ± 20%, with two scattering windows for each peak (6% for 75 keV and 10% for 48 keV), as illustrated in Fig. 1.

For activity measurements, a VIK-202 IBC dose calibrator was employed, calibrated to ^161^Tb in a 20 mL glass vial. When preparing phantoms, 4 g/L of DTPA was used to inhibit activity from binding to the inner walls of the phantom.

### Planar sensitivity and penetration

Planar sensitivity and penetration were measured using a Petri dish (∅= 15 cm) filled with a solution containing 100 MBq of ^161^Tb (*V* = 85 mL). Acquisitions were performed at ten source-to-collimator distances (1, 2, 5, 10, 15, 20, 25, 30, 35, and 40 cm), with the detectors in H-mode, using a 1024 × 1024 matrix (0.60 × 0.60 mm pixels) and a 180-s acquisition time.

The fraction of penetration (*PF*) was determined according to the NEMA standard [13], i.e., by fitting the recorded count rate in a small central region-of-interest (ROI), ∅ = 9 cm, to an exponential function with an added constant term. The *PF* was then determined as the fraction explained by the exponential term, as follows:

1 $$\:PF=\frac{{c}_{1}{e}^{-{c}_{2}{d}_{i}}}{{c}_{0}+{c}_{1}{e}^{-{c}_{2}{d}_{i}}}$$

where *c*_*0*_, *c*_*1*_ and *c*_*2*_ are fitting parameters and *d*_*i*_ is the source-to-collimator distance. The planar sensitivity was determined as the total recorded count rate per unit activity at *d* = 10 cm.

### Dead time

Dead-time measurements were performed intrinsically, i.e., with the collimators removed, using an array of ^161^Tb point sources. Measurements were performed one detector at a time (i.e., in single-head mode), with the detectors positioned upright and parallel to each other, with the active detector facing the wall of the examination room. The second detector head was removed during each acquisition (i.e., positioned behind the active detector) to allow for a homogeneous irradiation of the field-of-view (FOV).

Each source consisted of 10–30 µL ^161^Tb-DTPA in a plastic test tube (*n* = 11, *A* = 30–90 MBq). The sources were added one by one to a paper stand, located 2 m from the detector surface and aligned with the center of the detector’s FOV, resulting in an activity measurement range of 30–590 MBq.

As dead time is primarily associated with the total recorded count rate (i.e., the wide-spectrum count rate), five adjacent photopeak windows were employed, encompassing the entire available spectrum (18–690 keV): 35 keV ± 50%, 70 keV ± 20%, 140 keV ± 37.5%, 308 keV ± 37.5%, and 556 keV ± 24%.

Acquisitions were conducted until a total of 4,000,000 counts was reached, or for a minimum duration of 10 s (applicable to *A* > 200 MBq). A 256 × 256 matrix (2.4 × 2.4 mm pixels) was used, along with standard clinical acquisition settings. The background count rate was measured and accounted for. The procedure was repeated after 27 days, then with an activity range of 2–40 MBq.

To quantify the dead-time losses, the true wide-spectrum count rate (*R*_*T*_) was determined via linear extrapolation of the observed wide-spectrum count rate (*R*_*O*_) in the low-count-rate region (*R*_*O*_ < 93.3 kcps), where the data closely followed a linear relationship. The fractional dead-time loss (*ω*) was defined as:

2 $$\:\omega\:=1-\frac{{R}_{O}}{{R}_{T}}$$

To further characterize the count rate behavior, *R*_*O*_ was modeled as a function of *R*_*T*_ using Sorensen’s paralyzable model [14], applying a non-linear least square fit in MATLAB:

3 $$\:{R}_{O}\left({R}_{T}\right)={R}_{T}\bullet\:{e}^{-{R}_{T}\tau\:}$$

The dead-time constant *τ* was then extracted. Each detector was analyzed separately, and only *R*_*O*_ values below the observed saturation level were included.

### SPECT phantom evaluation

To assess the SPECT count-rate performance and predict the activities levels at which dead time could become significant, a cylindrical phantom (*V* = 6.28 L) with uniform activity (*A* = 343 MBq) was used. Measurements were carried out using both LEHR and MELP, with the ^161^Tb as well as the whole-spectrum window settings, using a 256 × 256 matrix, 60 projections, 30 s per projection, and clinical default settings.

From this, the extrinsic projection count rate per unit activity at different views (*θ*) could be determined as:

4 $$\:S\left(\theta\:\right)=\frac{R\left(\theta\:\right)}{A}$$

Where *R*(*θ*) is the observed wide-spectrum count rate and *A* is the phantom activity.

Based on this, a range of critical activities (*A*_*crit*_), corresponding to the dead-time losses observed in our intrinsic dead-time measurements, were estimated as follows:

5 $$\:{A}_{crit}=\frac{{R}_{O}}{1-\omega\:}\bullet\:\frac{1}{S\left(\theta\:\right)\:}$$

where *R*_O_ is the observed count rate producing a certain fractional dead-time loss $\:\omega\:$. *A*_*crit*_ was determined for *R*_*O*_ values associated with total saturation and the onset of non-linearity.

Furthermore, fractional dead-time losses for different hypothetical activity levels *A*_*crit*_ were derived from the model *R*_*O*_ (*R*_*T*_), by combining Eqs. (2–4), as:

6 $$\:\omega\:=1-{e}^{-{A}_{Crit}\bullet\:\tau\:\bullet\:S\left(\theta\:\right)}$$

In both Eqs. (5) and (6), the minimum, median, and maximum observed *S*(*θ*) were employed.

### Patient evaluation

To map out the count rate to be expected in post-therapeutic patient ^161^Tb imaging, data from eight patients treated with [^161^Tb]Tb-DOTA-LM3 were analyzed. The patients received a 1-GBq test injection of [^161^Tb]Tb-DOTA-LM3 as part of the BETA PLUS Phase 0a clinical trial (NCT05359146), and were imaged 3, 24, 72 and 168 h post injection (h p.i.) using the prescribed SPECT/CT protocol and a LEHR collimator. Two bed positions were used: the first encompassing shoulders to mid-abdomen, and the second ranging from mid-abdomen to the upper thighs, including the pelvic region.

The total recorded count rate per projection was extracted for both photopeak windows for each imaging time point. The wide-spectrum count rate was determined based on the count rate recorded in the 75 keV window (using the median 75 keV to whole-spectrum fraction observed the SPECT phantom measurements).

## Results

The planar in-air sensitivity for the 75 keV window was 15.7 cps/MBq for LEHR and 18.5 cps/MBq for MELP. At 48 keV, the sensitivity increased to 44.4 cps/MBq for LEHR and 67.9 cps/MBq for MELP, corresponding to a 2.8-fold and 3.7-fold increase, respectively.

**Fig. 2:**
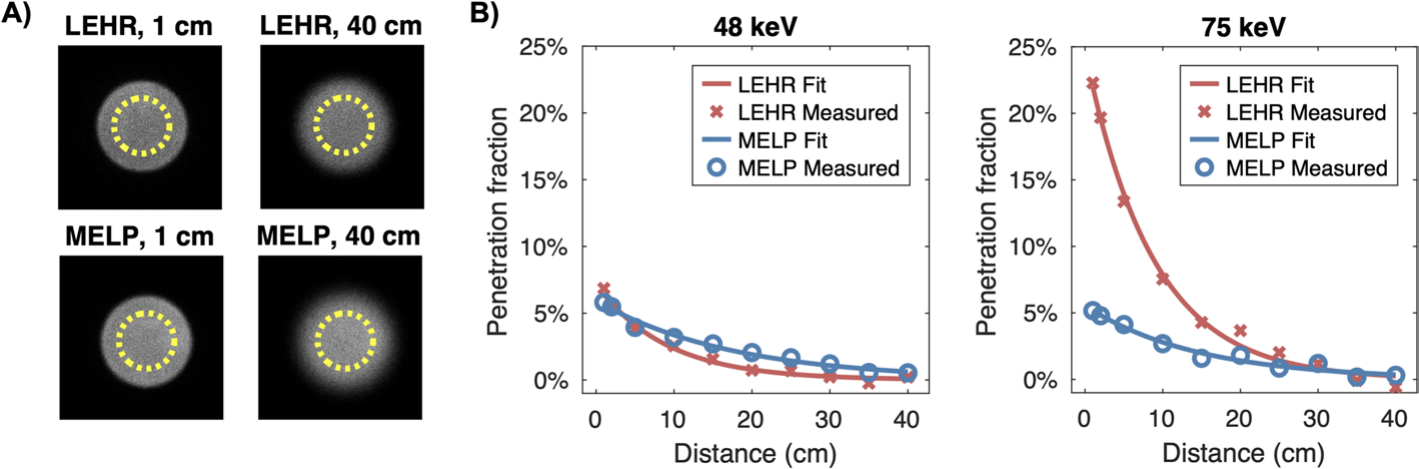
The penetration fraction (PF) as a function of source-to-collimator distance for LEHR and MELP at 48 and 75 keV (**B**), accompanied by selected planar images of the Petri dish and the employed ROI (**A**)

The highest PFs were observed for LEHR with the 75 keV window, reaching 7.5% and 22% at 10 cm and 1 cm, respectively. For all other window-collimator combinations, PFs remained ≤ 3.2% at 10 cm and ≤ 6.9% at 1 cm (see Fig. 2).

**Fig. 3:**
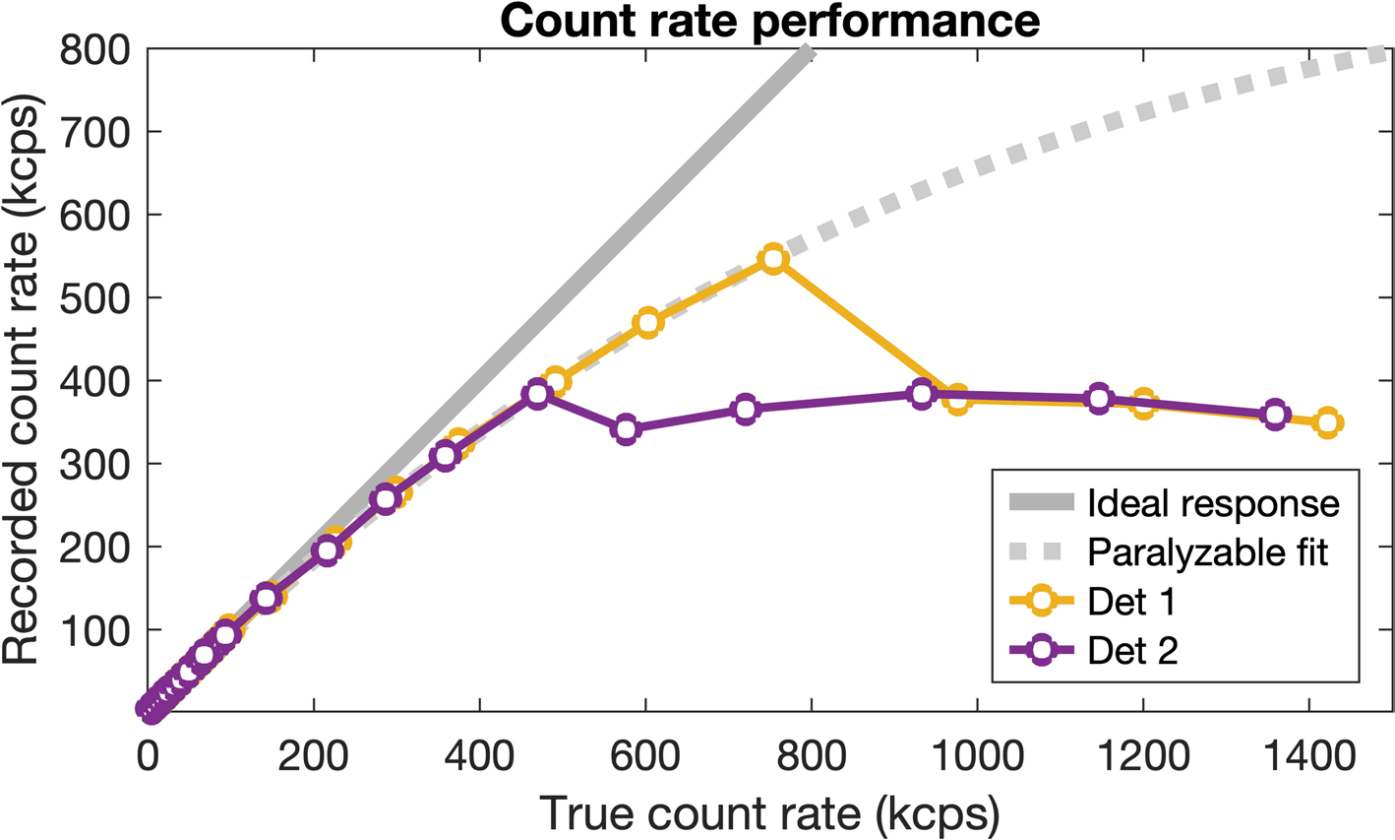
Recorded count rate as a function of true count rate for the intrinsic dead-time measurements

Concerning the intrinsic dead-time measurements, the increase in the wide-spectrum count rate became non-linear at approximately 100 kcps (99.2 and 93.3 kcps for Detector 1 and 2, respectively). Saturation occurred at *R*_O_ >384 kcps for Detector 1, and *R*_O_ >546 kcps for Detector 2 (see Fig. 3), manifesting as a sharp decline in count rate. At the point of saturation, the dead-time loss was 18% and 28% for Detector 1 and 2, respectively. The dead-time constant was estimated to 0.42 µs for both detectors (0.422 ± 0.008 µs and 0.421 ± 0.034 µs for Detector 1 and 2, respectively).

**Fig. 4:**
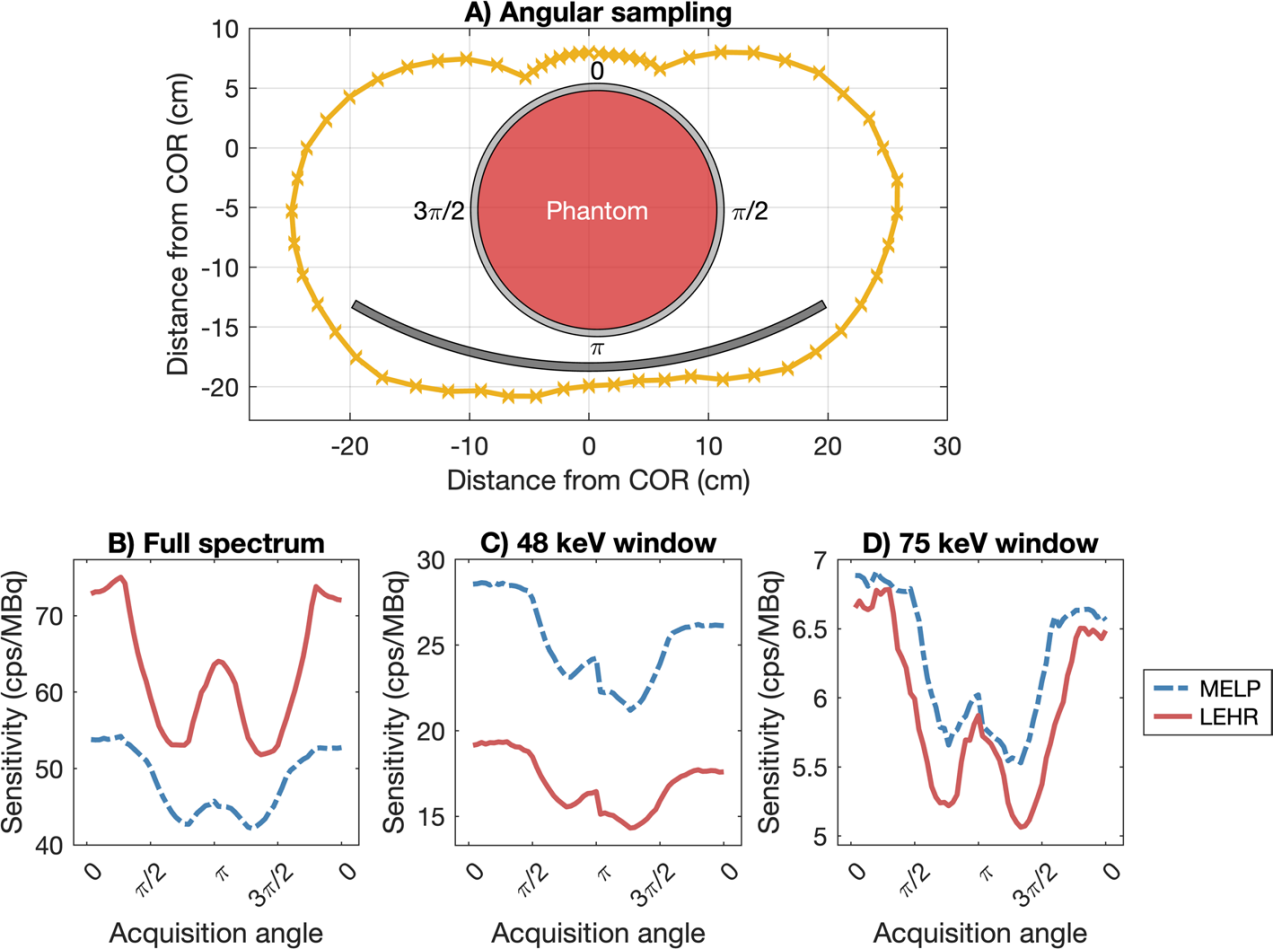
Evaluation of SPECT projection count rate. In (**A**), the angular sampling of the acquisitions (i.e., the detector paths) relative to the phantom and patient table is shown. (**B**), (**C**), and (**D**) display the corresponding count rate per unit activity for the whole spectrum, the 48 keV window, and the 75 keV window, respectively

For the SPECT acquisition of the cylindrical phantom (Fig. 4A), the median projection count rate, *S*(*θ*), was 61.8 cps/MBq with LEHR (range: 51.8–75.0 cps/MBq) and 48.1 cps/MBq with MELP (range: 42.2–54.2 cps/MBq) for the total spectrum (Fig. 4B). For the 75 keV window, the projection count rate ranged from 5.06 to 6.78 cps/MBq for LEHR (8.81–10.2% of the total) and from 5.53 to 6.91 cps/MBq for MELP (12.4–13.5% of the total) (Fig. 4D). At 48 keV, the projection count rate ranged from 14.3 to 19.4 cps/MBq for LEHR (23.6–31.2% of the total) and from 21.2 to 28.7 cps/MBq for MELP (49.4–55.3% of the total) (Fig. 4C).

Table 1 presents the predicted dead-time losses for the cylindrical SPECT phantom, derived from the *R*_*T*_(*R*_*O*_) model at different activity levels, as determined using Eq. (6). The critical activities, predicted with Eq. (5), are summarized in Table 2. Dead-time effects are expected at in-field activities above 1.2 GBq for LEHR and 1.7 GBq for MELP, with total saturation predicted at activities exceeding 6.3 GBq for LEHR and 8.7 GBq for MELP. Note that these thresholds correspond to the projections with the highest observed projection count rates, i.e., maximum *S*(*θ*), and the detector with the poorest performance (Detector 2).

**Table 1:** Estimated dead-time losses, as predicted by the *R*_*T*_(*R*_*O*_) model, at different given in-field ^161^Tb activities

	Fractional dead-time loss, $\:\omega\:$ (%)	
In-field activity (GBq)	MELP	LEHR
1	1.8–2.2	2.2–3.1
2	3.5–4.4	4.3–6.0
3	5.2–6.5	6.3–8.9
4	6.9–8.5	8.4–12
5	8.5–11	10–14
6	10–13	12–17
7	12–14	14–20*
8	13–16	16–22*
9	15–18	18–24*
10	16–20*	20*–27*

*= Above the observed saturation threshold

**Table 2:** Estimated critical in-field activity (min-max, median) levels for the cylindrical SPECT Phantom

	Detector 2		Detector 1	
Collimator	Non-linear (GBq) (*R*_O_ = 93.3 kcps)	Saturation (GBq) (*R*_O_ = 384 kcps)	Non-linear (GBq) (*R*_O_ = 99.2 kcps)	Saturation (GBq) (*R*_O_ = 546 kcps)
LEHR	1.2–1.8 (1.5)	6.3–9.1 (7.6)	1.3–1.9 (1.6)	10–15 (12)
MELP	1.7–2.2 (1.9)	8.7–11 (9.8)	1.8–2.4 (2.1)	15–18 (16)

**Fig. 5:**
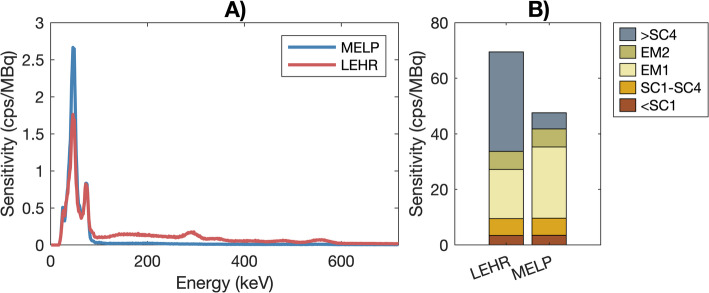
Recorded energy spectra of the SPECT cylinder phantom acquired at θ = 0°, corresponding to the minimal phantom-to-collimator distance (**A**), and the corresponding sensitivity in different energy regions (**B**), both within and outside the defined windows (Fig. 1)

In Fig. 5, complete energy spectra corresponding to the 0° acquisition are shown (A), along with bar plots representing the fraction of counts recorded in different energy regions (B). For LEHR, 51.6% of all counts are recorded above the highest energy window (> SC4). For MELP, the corresponding fraction is 12.2%.

For the patients treated with 1 GBq of [^161^Tb]Tb-DOTA-LM3, the recorded count rates varied significantly depending on the patient and bed position (see Suppl. Table 2). Assuming a 75 keV window fraction of 9.47% (as observed in the cylindrical phantom), the median wide-spectrum count rate at 3 h p.i. was 19.1 kcps per injected GBq, with a range of 6.79–39.0 kcps/GBq, corresponding to a maxmimum dead-time loss of 1.7%, according to the derived model.

## Discussion

While the feasibility of high-quality ^161^Tb SPECT imaging has been demonstrated [15, 16], more detailed descriptions of the response in γ-camera imaging is lacking. In this study, we evaluated sensitivity, septal penetration, and count rate behavior for two different energy windows (48 keV ± 20% and 75 keV ± 10%) and two collimators (LEHR and MELP) using a Siemens Symbia Intevo SPECT system. These investigations are particularly relevant as the system is currently used in two clinical trials involving ^161^Tb-based radiopharmaceuticals (BETA PLUS; NCT05359146 and PROGNOSTICS; NCT06343038) [7, 10]. As future study phases will involve increased administered activities of ^161^Tb, we aimed to investigate dead-time effects in anticipation of potential limitations.

### Sensitivity and penetration

The primary imageable emissions of ^161^Tb are relatively low in energy, making them more susceptible to in-patient attenuation. Even so, the sensitivity for ^161^Tb remains reasonable. In this study, the planar in-air sensitivity was determined to 15.7 and 18.5 cps/MBq for LEHR and MELP, exceeding values reported for similar systems using 208 keV ^177^Lu with a medium-energy collimator (e.g., 9.36 and 10.1 cps/MBq) [17, 18], likely due to the higher detection efficiency of NaI(Tl) at lower photon energies. When attenuation is present, the count rate per unit activity in the ^161^Tb 75 keV window (~ 6 cps/MBq, see Fig. 4) appears roughly comparable to that of the 208 keV window for ^177^Lu (~ 7 cps/MBq [17], deduced from tabular data).

At 48 keV, sensitivity is even higher than at 75 keV, partly, as this window includes not only the 49 keV γ-peak but also several intense X-rays (e.g., 45.2 keV, 6.28%; 46.0 keV, 11.2%) [11]. While quantification at this energy presents additional challenges—such as reduced recovery [16] and down-scatter contributions from the 75 keV peak—sensitivity remains favorable, making this window a potential asset at late-time point imaging. Overall, ^161^Tb demonstrates good potential for clinical imaging with reliable counting statistics.

For the 75 keV window, the in-air sensitivity (i.e., as measured at 10 cm) was similar for both collimators, yet slightly higher for MELP. However, at larger distances—where scatter and penetration are minimized—the MELP sensitivity remained higher than for LEHR (18.1 vs. 13.3 cps/MBq, Suppl. Table 1), highlighting that the primary count rate is indeed higher with medium-energy collimation.

A more notable difference between the collimators was noted for the 48 keV window (e.g., Fig. 4). At 10 cm, the planar sensitivity at 48 keV was 3.7 times higher than at 75 keV for MELP, whereas for LEHR, the increase was only 2.8-fold. This difference can, in part, be attributed to penetration effects; the contribution from down-scattered photons is higher for LEHR at 75 keV, which leads to a lower 48 keV-to-75 keV sensitivity ratio. At the maximum source-to-collimator distance—where the scatter contribution is minimized—the relationship between the two windows becomes more similar (see Suppl. Table 1), supporting the argument above. However, even at 40 cm, the 48 keV-to-75 keV sensitivity ratio remains slightly higher for MELP compared to LEHR (3.2 and 3.6, respectively, see Table 1, Suppl). One possible explanation is that the use of the MELP collimator increases the production of characteristic X-rays, which contribute more to the 48 keV window. This hypothesis remains to be verified.

Regarding septal penetration, the greatest degradation was observed for LEHR with the 75-keV window, reaching a peak PF of 22% at a 1-cm source-to-collimator distance in the planar Petri dish measurements (see Fig. 2). The effects of penetration were also evident in the SPECT measurements. At 75 keV, the count rate for projections acquired at the closest source-to-collimator distance was 34% higher than those at the farthest (5.06 vs. 6.78 cps/MBq), while for MELP, the increase was 25% (5.53 vs. 6.91 cps/MBq), see Fig. 4. Penetration poses challenges both qualitatively and quantitatively; visually, it can introduce streak artifacts and reduce image contrast, while quantitatively, it leads to unwanted contributions within the photopeak window, affecting measurement accuracy. Although methods of scatter correction, e.g., the triple-energy window (TEW) method, can mitigate these effects to some extent, minimizing penetration at the acquisition stage remains the preferable approach for ensuring reliable quantification.

The impact of penetration was evident when examining the energy spectra acquired (see Fig. 5). For LEHR, penetrating peaks are noticeable at, e.g., 292 and 550 keV. Although the yields of these emissions are low (0.058% and 0.036%, respectively) [11], they—along with other low-yield higher-energy emissions—still contribute significantly. For LEHR, 51.6% of all counts are recorded above the highest energy window, whereas for MELP the corresponding fraction is only 12.2%. This highlights how penetration is linked to dead time; penetration and scatter will result in unwanted counts, elevating the wide-spectrum count rate, thus, promoting dead time. Specifically, the wide-spectrum count rate was 51.8–75.0 cps/MBq for LEHR and 42.2–54.2 cps/MBq for MELP. Assuming dead-time losses are directly proportional to the wide-spectrum count rate, using MELP would increase the system’s measurement capability by 23–38%.

### Dead time

In this study, dead-time was estimated intrinsically using a set of ^161^Tb point sources, measured at two separate occasions; a methodology similar to that described by Hjellström et al. [19]. This approach offers a quick and practical way of estimating the dead time, as opposed to extrinsic phantom measurements, which require more time and higher activity.

We assume that the measured dead time is applicable in a clinical setting, where collimators and scattering material will alter the energy distribution of detected photons. It is generally accepted that dead time is primarily dictated by the total count rate across the entire spectrum, which is what we have considered. Other authors have suggested that, when considering only the total count rate, dead time should be relatively independent of photon energy and nuclide [20, 21]. Also, the fractional dead-time loss within a selected photopeak window should be close to proportionate to that of the entire spectrum, unless pulse pile-up effects are severe [22].

This said, primary photon energy appears to have some influence on dead time. However, no exact relationship between dead time and energy has been described in the literature. Desy et al. and Frezza et al. measured dead time on the same Siemens Symbia T6 camera (a model similar to the device used in this work) using ^177^Lu and ^99m^Tc, respectively, and reported values of 0.55 µs and 0.49 µs [17, 21]. In this study, we obtain 0.42 µs, suggesting a trend of increasing dead time with higher photon energy. This would be reasonable, as higher energy results in higher pulse amplitudes, potentially increasing the demand on the pulse height analyzer (PHA). Other components within the γ-camera could also impose limitations, but we do not have detailed insight into which components set the constraints or how they influence dead time.

Measurement geometry has also been shown to affect dead time. Heemskerk and Defrise conducted a comprehensive study of dead time using ^99m^Tc [23]. They observed a reduction in the wide-spectrum dead time when a collimator was applied: 1.25–1.30 µs intrinsically with point sources, 0.99–1.07 µs extrinsically with point sources, and 0.87–0.94 µs extrinsically with a phantom including scatter. One possible explanation is, once again, that the collimator and scattering material reduce the average photon energy, making pulses easier to process by the PHA, thus, lowering the dead time. This suggests that the actual dead time for ^161^Tb in a clinical SPECT/CT setting—including collimators and patient scatter—could be lower than the intrinsic value estimated here. However, these results apply to ^99m^Tc, which has a single primary energy of 140 keV. For ^161^Tb, with its broader energy distribution, the effect is expected to be less pronounced. Still, this highlights a limitation of our approach: the dead-time constant obtained from intrinsic measurements may overestimate the dead time experienced during clinical imaging with a collimated setup. These potential discrepancies should be kept in mind when applying the threshold estimates reported here to real-world imaging.

McIntosh et al. determined the ^161^Tb SPECT sensitivity in reconstructed images (corrected for attenuation and scatter) to be 14.2 cps/MBq for the 75 keV window with a LEHR collimator, based on measurements in the 250–2000 MBq range, using a similar system [15]. This aligns well with our planar sensitivity of 15.7 cps/MBq for LEHR. While the methodologies are not directly comparable—McIntosh et al. used dual-head acquisition with full correction, while we report single-head planar sensitivity—the agreement is consistent with the theoretical expectation that properly corrected reconstructed sensitivity should approximate planar sensitivity. Moreover, McIntosh et al. measured the sensitivity across a broader activity range (70–4990 MBq) using a NEMA IEC Body Phantom and a LEHR collimator, and reported that uncorrected dead-time effects became apparent above 2 GBq. However, they also observed an activity-dependent variation in sensitivity across the entire examined range, attributing the higher sensitivity at low activities to reconstruction-related issues. At 5 GBq, they estimated dead-time losses of approximately 20%. The discrepancy between their findings and ours could be due to camera-specific differences or variations in measurement geometry. Nonetheless, their results support our predicted critical activity levels, reinforcing that dead-time effects occur at significantly lower activities for ^161^Tb compared to ^177^Lu.

It is worth noting that Siemens systems generally appear to perform well in terms of dead time. Desy et al. measured a dead time of 0.49 µs for ^99m^Tc with a Siemens camera, whereas two GE cameras using the same method showed considerably higher values, 1.74 µs [21]; hence, the critical activity levels predicted in this work could be significantly lower with a different system.

One important factor to consider is the dead-time saturation threshold. While other modern systems have been shown to follow a more purely paralyzable behavior [19, 21], the system examined in this study exhibits a gradual reduction in count rate—well described by the Sorenson model—followed by a sharp drop. This occurred at distinctly different levels for the two detector heads (384 and 546 kcps, respectively), a phenomenon also observed by others [21]. Desy et al. also reported significant differences in saturation levels depending on which detector heads were active, with saturation thresholds of 480 and 290 kcps for dual-detector acquisition, while in single-detector mode, they measured 700 and 475 kcps [21]. A similar pattern was confirmed in another study using the same camera model [18]. While dead-time behavior below the saturation threshold appears independent of detector activation, this has important implications for our study: since our measurements were performed in single-head acquisition mode, the predicted critical saturation activities may be significantly lower.

### Clinical implications

Concerning dead time, the patient count rates observed for 1 GBq of [^161^Tb]Tb-DOTA-LM3 were well below the range where dead-time effects are expected (Suppl. Table 2), with an estimated median whole-spectrum count rate of ~ 20 kcps at 3 h p.i. However, at therapeutic activity levels, dead-time effects could become significant. Assuming a linear relationship between count rate and activity, an administered activity of 7.4 GBq would correspond to a median count rate of approximately 150 kcps and a maximum of around 300 kcps at 3 h p.i., leading to estimated dead-time losses of 6.5% and 13.6%, respectively. For comparison, Uribe et al. reported typical whole-spectrum count rates of 50–70 kcps for patients treated with 7.4 GBq of ^177^Lu at 4 h p.i., which is about one third of the values observed in our study [21].

It should be noted that our dead-time estimates are based on measurements with a cylindrical SPECT phantom. In a clinical setting, patient-wide spectrum count rates may be even higher due to increased scattering. Additionally, patient positioning plays a crucial role. For the patients in this study, high-activity regions (e.g., liver, kidneys, and spleen) were distributed across two FOVs. A single FOV centered on the abdomen would result in even higher count rates and increased dead time. Moreover, while we assume a linear relationship between administered activity and count rate, nonlinear effects in biodistribution and kinetics at higher activity levels cannot be excluded and may influence the actual count rate.

In terms of recommended settings for clinical imaging, the findings in this study suggest that medium-energy collimation combined with a 75 keV ± 10% energy window offers more robust prerequisites for quantification, as it reduces degradation from septal penetration and dead time. This contrasts with current literature, which supports the use of low-energy collimation [15, 16]. That said, centers with access to advanced reconstruction methods and the ability to measure and correct for dead-time losses may achieve sufficient accuracy even with low-energy collimation, benefiting from improved spatial resolution. Ultimately, optimal imaging parameters—including collimator type and energy window—may need to be adapted based on the specific system and reconstruction capabilities available at each center. Regardless, for early time-point imaging with ^161^Tb at clinical activity levels, dead-time effects should be closely monitored and possibly accounted for.

In a clinical setting, the 48 keV energy window may offer advantages for delayed acquisitions due to its higher sensitivity. However, this window is more susceptible to scatter degradation—including 75 keV down-scatter, characteristic X-rays, and increased coherent scattering—which can vary significantly between patients. Obtaining good quantitative accuracy would therefore likely require more a robust scatter correction, ideally Monte Carlo-based.

Based on our preliminary experience, a practical strategy could be to reconstruct both energy windows separately with individually optimized parameters. If agreement between the two windows is observed at earlier time points, the 48 keV window could be used to replace or complement the 75 keV estimate at later time points, when count rates are low. Defining a count threshold within the VOI below which the 75 keV window becomes unreliable may offer a practical trigger for switching to the 48 keV window.

It should also be noted that dead-time effects may be more pronounced at 48 keV at high activities, due to pulse pile-up. Additionally, the attenuation correction at this energy is likely more sensitive to spatial misalignments, e.g., misregistration between SPECT and CT due to patient movements, which could introduce larger errors compared to the 75 keV window.

## Conclusions

The decay scheme of ^161^Tb is particularly complex, involving numerous photon emissions across a broad energy range. Our findings emphasize the need for optimized acquisition protocols, particularly regarding collimator selection and dead-time correction, to ensure accurate dosimetry in γ-camera imaging when using ^161^Tb.

At 75 keV, sensitivity was comparable to that reported for the 208 keV window of ^177^Lu, regardless of collimator choice. At 48 keV, sensitivity was significantly higher, which could be advantageous for late-time points if associated challenges are addressed.

Dead-time effects should be carefully considered in ^161^Tb imaging. The wide-spectrum count rate can be up to three times higher than reported for ^177^Lu, increasing the risk of dead-time losses. The use of low-energy collimators enhances visual image quality but also increases penetration and scatter, potentially impairing quantification and further elevating count rates. Thus, a trade-off is required in collimator selection. At clinical activity levels (e.g., 7.4 GBq), dead time should be closely monitored to ensure reliable quantification.

## Electronic supplementary material

Below is the link to the electronic supplementary material.

Supplementary Material 1

## Data Availability

The datasets used and/or analyzed during the current study are available from the corresponding author on reasonable request.

## Abbreviations

DTPA: Diethylenetriaminepentaacetic acid
ROI: Region of interest
FOV: Field-of-view
LEHR: Low-energy high-resolution
MELP: Medium-energy low-penetration
PF: Penetration fraction
PHA: Pulse height analyzer
SPECT: Single Photon Emission Computed Tomography
CT: Computed Tomography
