# Supplementary material for: Bridging physics and practice: evaluating sensitivity, septal penetration, and detector dead time in terbium-161 gamma-camera imaging
Source: EJNMMI Phys. 2025 Aug 28;12:81. doi: 10.1186/s40658-025-00792-x (PMC12390893; doi:10.1186/s40658-025-00792-x)
Supplement: Supplementary file 1 — Supplementary Material 1 [file 40658_2025_792_MOESM1_ESM.docx]

**Supplements**

Suppl Table 1: Sensitivity for LEHR and MELP at different source-to-collimator distances.

|  | LEHR (cps/MBq) | |  | MELP (cps/MBq) | |  |
| --- | --- | --- | --- | --- | --- | --- |
| Distance (cm) | 75 keV | 48 keV | Ratio | 75 keV | 48 keV | Ratio |
| 1 | 16.6 | 45.7 | 2.75 | 18.8 | 69.3 | 3.69 |
| 2 | 16.6 | 45.4 | 2.73 | 18.8 | 69.1 | 3.68 |
| 5 | 16.3 | 44.9 | 2.75 | 18.7 | 68.5 | 3.66 |
| 10 | 15.7 | 44.4 | 2.83 | 18.5 | 67.9 | 3.67 |
| 15 | 15.1 | 43.9 | 2.91 | 18.4 | 67.4 | 3.66 |
| 20 | 14.6 | 43.4 | 2.97 | 18.4 | 67.0 | 3.64 |
| 25 | 14.1 | 43.1 | 3.06 | 18.2 | 66.7 | 3.66 |
| 30 | 13.8 | 42.7 | 3.09 | 18.2 | 66.4 | 3.65 |
| 35 | 13.5 | 42.4 | 3.14 | 18.1 | 66.0 | 3.65 |
| 40 | 13.3 | 42.3 | 3.18 | 18.1 | 65.8 | 3.64 |

Suppl Table 2: The median projection count rate, along with the the minimum–maximum range (kcps), for each imaging time-point (3, 24, 72 and 168 h p.i.) and photopeak window (48 keV ± 20% and 75 keV ± 10%) for patients treated with 1 GBq of [^161^Tb]Tb-DOTA-LM3, as part of the BETA PLUS Phase 0a trial (NCT05359146).

|  |  |  | Patient | | | | | | | |
| --- | --- | --- | --- | --- | --- | --- | --- | --- | --- | --- |
| Window | Time  (h p.i.) | Bed | 1 | 2 | 3 | 4 | 5 | 6 | 7 | 8 |
| 75 keV | 3 | 1 | 1.91  (1.61–2.53) | 1.44  (0.96–1.81) | 2.34  (1.89–2.86) | 2.08  (1.39–2.67) | 3.09  (1.18–3.84) | 1.57  (1.23–1.96) | 1.90  (1.18–2.53) | 1.83  (0.97–2.35) |
|  |  | 2 | 1.91  (1.47–2.19) | 1.94  (1.34–3.02) | 1.67  (1.17–2.29) | 1.72  (1.15–2.48) | 1.33  (0.67–1.78) | 1.22  (0.88–1.74) | 1.59  (1.02–2.43) | 1.91  (1.1–2.51) |
|  | 24 | 1 | 1.60  (1.26–2.09) | 0.96  (0.66–1.29) | 1.79  (1.45–2.24) | 1.65  (1.06–2.16) | 2.41  (0.94–3.01) | 1.11  (0.90–1.35) | 1.3  (0.87–1.87) | 2.11  (0.99–2.85) |
|  |  | 2 | 1.57  (1.16–1.80) | 1.76  (1.26–2.82) | 1.21  (0.85–1.70) | 1.08  (0.72–1.48) | 1.12  (0.54–1.51) | 0.95  (0.68–1.31) | 1.18  (0.79–1.89) | 1.09  (0.67–1.53) |
|  | 72 | 1 | 1.18  (0.92–1.53) | 0.53  (0.37–0.71) | 1.23  (1.01–1.49) | 0.99  (0.70–1.30) | 0.24  (0.17–0.31) | 0.73  (0.59–0.89) | 0.76  (0.58–1.10) | 1.68  (0.75–2.36) |
|  |  | 2 | 1.06  (0.80–1.25) | 1.23  (0.91–1.98) | 0.75  (0.54–1.07) | 0.82  (0.55–1.10) | 1.47  (0.63–1.98) | 0.60  (0.44–0.85) | 0.83  (0.6–1.35) | 0.60  (0.39–0.87) |
|  | 168 | 1 | 0.57  (0.43–0.79) | 0.34  (0.25–0.44) | 0.60  (0.51–0.78) | 0.40  (0.28–0.54) | 0.35  (0.15–0.46) | 0.30  (0.23–0.36) | 0.18  (0.13–0.23) | 0.85  (0.35–1.19) |
|  |  | 2 | 0.55  (0.38–0.63) | 0.36  (0.20–0.59) | 0.28  (0.20–0.41) | 0.30  (0.21–0.42) | 0.28  (0.14–0.39) | 0.29  (0.23–0.41) | 0.39  (0.28–0.64) | 0.35  (0.21–0.5) |
| 48 keV | 3 | 1 | 5.36  (4.23–6.74) | 4.41  (2.75–5.67) | 7.19  (5.70–9.26) | 6.30  (4.04–8.73) | 10.05  (3.48–13.35) | 5.29  (3.92–6.47) | 5.88  (3.44–8.44) | 5.74  (2.78–7.92) |
|  |  | 2 | 5.12  (3.97–6.15) | 5.81  (4.22–9.86) | 5.04  (3.29–7.51) | 5.29  (3.27–8.18) | 4.16  (1.89–5.93) | 4.03  (2.74–6.12) | 4.88  (3.00–8.14) | 6.11  (3.34–8.57) |
|  | 24 | 1 | 4.40  (3.26–5.40) | 2.92  (1.86–4.00) | 5.50  (4.55–7.30) | 5.06  (3.2–7.18) | 7.88  (2.82–10.39) | 3.67  (2.83–4.59) | 3.87  (2.47–6.22) | 6.69  (2.86–9.66) |
|  |  | 2 | 4.10  (3.05–4.87) | 5.23  (3.89–9.21) | 3.64  (2.32–5.46) | 3.31  (2.00–4.88) | 3.56  (1.59–5.07) | 3.08  (2.12–4.52) | 3.55  (2.26–6.31) | 3.31  (1.95–5.17) |
|  | 72 | 1 | 3.11  (2.30–3.86) | 1.52  (1.00–2.16) | 3.80  (3.18–4.90) | 2.99  (2.00–4.3) | 0.61  (0.39–0.85) | 2.42  (1.99–2.97) | 2.26  (1.70–3.63) | 5.2  (2.17–7.80) |
|  |  | 2 | 2.68  (1.97–3.28) | 3.65  (2.79–6.48) | 2.26  (1.48–3.46) | 2.46  (1.55–3.56) | 4.98  (1.87–6.71) | 1.95  (1.37–2.97) | 2.49  (1.76–4.57) | 1.72  (1.07–2.77) |
|  | 168 | 1 | 1.38  (0.99–1.75) | 1.00  (0.73–1.37) | 1.82  (1.55–2.56) | 1.17  (0.78–1.72) | 1.09  (0.43–1.52) | 0.95  (0.71–1.21) | 0.50  (0.33–0.69) | 2.55  (0.98–3.8) |
|  |  | 2 | 1.21  (0.83–1.46) | 1.01  (0.57–1.89) | 0.78  (0.50–1.26) | 0.90  (0.58–1.32) | 0.86  (0.39–1.28) | 0.95  (0.72–1.37) | 1.16  (0.81–2.15) | 0.98  (0.56–1.52) |
